# Epileptic spasms in the acute phase of pediatric anti-N-methyl-d-aspartate receptor encephalitis: a case series

**DOI:** 10.3389/fimmu.2026.1807048

**Published:** 2026-07-07

**Authors:** Pingping Tian, Guiling Liu, Wandong Hu, Wenchao Zhang, Fen Zhao, Hongwei Zhang

**Affiliations:** Department of Pediatric Neurology, Children’s Hospital Affiliated to Shandong University/Jinan Children’s Hospital, Jinan, China

**Keywords:** acute phase, autoimmune encephalitis, children, epileptic spasms, N-methyl-D-aspartate receptor (NMDAR) antibody encephalitis

## Abstract

**Background:**

Anti-N-methyl-D-aspartate receptor encephalitis (NMDARE) is an autoimmune disorder characterized by a broad spectrum of neuropsychiatric symptoms. Epileptic seizures are among the most common clinical manifestations, occurring in approximately 25% of patients. However, epileptic spasms as a distinct seizure type in the acute phase of NMDARE have rarely been documented.

**Case presentation:**

We reported three pediatric patients who developed epileptic spasms during the acute phase of NMDARE. The cohort included two females and one male, with age at onset ranging from 9 months to 9 years. Epileptic spasms emerged within one month of disease onset and were accompanied by focal seizures in all cases. Electroencephalography (EEG) monitored frequent epileptic spasms in all three children. Brain magnetic resonance imaging (MRI) revealed bilateral frontal lobe lesions in two patients. Following combined immunotherapy of intravenous immunoglobulin plus methylprednisolone or therapeutic plasma exchange, one patient discontinued treatment, one experienced recurrence two years later, and one showed favorable recovery at the last follow-up of five months.

**Conclusion:**

This case series highlights epileptic spasms as a notable and less common clinical phenotype in the acute stage of NMDARE. Our findings broaden the recognized clinical spectrum of the NMDARE and offer practical insights for diagnosis and management in pediatric practice.

## 1 Introduction

N-methyl-D-aspartate receptor (NMDAR) antibody encephalitis (NMDARE) is the most common autoimmune encephalitis (AE) in pediatric population, accounting for 54% to 80% of all AE patients (1). With an incidence of approximately 0.17 per 100,000 children, NMDARE is a significant cause of acquired neurological morbidity. While the majority of pediatric patients achieve favorable outcomes with standard first-line immunotherapy (2, 3), the clinical course can be severe. During the acute or subacute phase of three months, mortality remains a concern in 5%-10% of patients (4), and approximately 25% could develop a refractory disease with prolonged and severe neurological deficits (5). Therefore, early identification of clinical features, especially those heralding a severe disease course, as well as the implementation of early intervention measures, are critical for improving the prognosis of patients, especially in tertiary care settings.

NMDARE typically presents with a broad spectrum of neuropsychiatric manifestations (6), including psychiatric and behavioral abnormalities, consciousness disturbances, epileptic seizures, involuntary movements, sleep disorders, and autonomic nerve disorders, with positive anti-NMDAR antibodies production (2). In acute phase, epileptic seizure might be the initial presenting symptom in a substantial number of cases (7). According to statistical data, over 80% of patients experience seizures during this period (8, 9). Furthermore, a prior study has showed seizure was more likely to recur in patients with severe conditions, such as tumor presence, status epilepticus (SE) development, or intensive care unit (ICU) admission in the acute phase (10). As a diffuse encephalitis, NMDARE can provoke diverse seizure types, with generalized tonic clonic seizures being the most frequently reported (10, 11). However, the occurrence of epileptic spasms as a novel manifestation of acute-phase NMDARE has been rarely reported to date (12). According to the ILAE 2017 operational classification (13), epileptic spasms are defined as sudden flexion, extension, or mixed extension- flexion of predominantly proximal and truncal muscles, with limited forms including grimacing, head nodding, or subtle eye movements; they frequently occur in clusters and can manifest at any age. Interictal EEG may show hypsarrhythmia, hypsarrhythmia-like patterns, or unremarkable findings, whereas ictal EEG manifestations are diverse, including voltage attenuation, high- amplitude biphasic slow waves, or spike- and- wave discharges.

Herein, we report three pediatric cases of acute NMDARE that presented with epileptic spasms. This case series aims to expand the recognized phenotypic spectrum of pediatric NMDARE and to provide clinicians with valuable insights for the timely identification and management of this distinct seizure semiology in the context AE.

## 2 Case presentation

Informed consents were signed by the participants or the legal representatives. The clinical information of the three cases was summarized in **Table 1**.

**Table 1:** The clinical information of the three patients.

Variable	Case 1	Case 2	Case 3
Age of onset	9 months	9 years 4 months	5 years 2 months
Sex	Male	Female	Female
The time from disease onset to first spasms seizure	24 days	21 days	16 days
Main clinical manifestations	Fever, status epilepticus, dysphoria→dyskinesia of right upper extremity, distortion of commissure, dysphagia, conscious disturbance	Epileptic seizure, aphasis, involuntary movement→status epilepticus, abnormal behavior, cognitive impairment, and conscious disturbance	Sleep disorders, abnormal mental and behavioral manifestations, epileptic seizures, involuntary movements→cognitive impairment, dysphagia, conscious disturbance, dyskinesia, distortion of commissure
Other seizure types	Focal seizure	Focal seizure, EPC	Focal seizure
Anti-NMDAR antibodies titer	Serum1:32; CSF 1:32	Serum1:100; CSF 1:10	Serum1:100; CSF 1:32
Detected pathogen	HSV-1 (CSF)	HSV-1 (serum)	HSV-1 (serum)
MRI	Bilateral frontal lobes, left parietal lobe	Normal	Bilateral frontal lobe, insular lobe, and left temporal lobe
mRS score	Not Applicable	Admission, mRS=5→Follow-up, mRS=0→Recurrence, mRS=3→ Follow-up, mRS=0	Onset, mRS=3→Peak, mRS=5→Follow-up, mRS=2
ASMs	LEV, TPM	PER, LEV, KD	PER, CLB, KD
Treatments	two rounds of IVIG (2g/kg), IVMP	Plasma exchange for 5 rounds, IVIG (2g/kg) for 2 rounds, IVMP (20mg/kg/d)	IVMP (20mg/kg/d); IVIG (2g/kg); Rituximab (375mg/m2) for 4 doses; Tocilizumab (150mg) for 1 dose; IVIG (1-2g/kg, once a month) for maintenance therapy
Follow-up	1 month	2 years	3 months
Outcomes	Abandoning treatment, then died	Recurrence	In recovery

EPC, epilepsia partialis continua; HSV-1, herpes simplex virus type 1; CSF, cerebrospinal fluid; MRI, magnetic resonance imaging; mRS, Modified Rankin Scale; ASMs, anti-seizure medications; LEV, levetiracetam; TPM, topiramate; PER, perampanel;KD, ketogenic diet; CLB, clobazam; IVMP, intravenous methylprednisolone; IVIG, intravenous immunoglobulin.

### 2.1 Case 1

**Figure 1:**
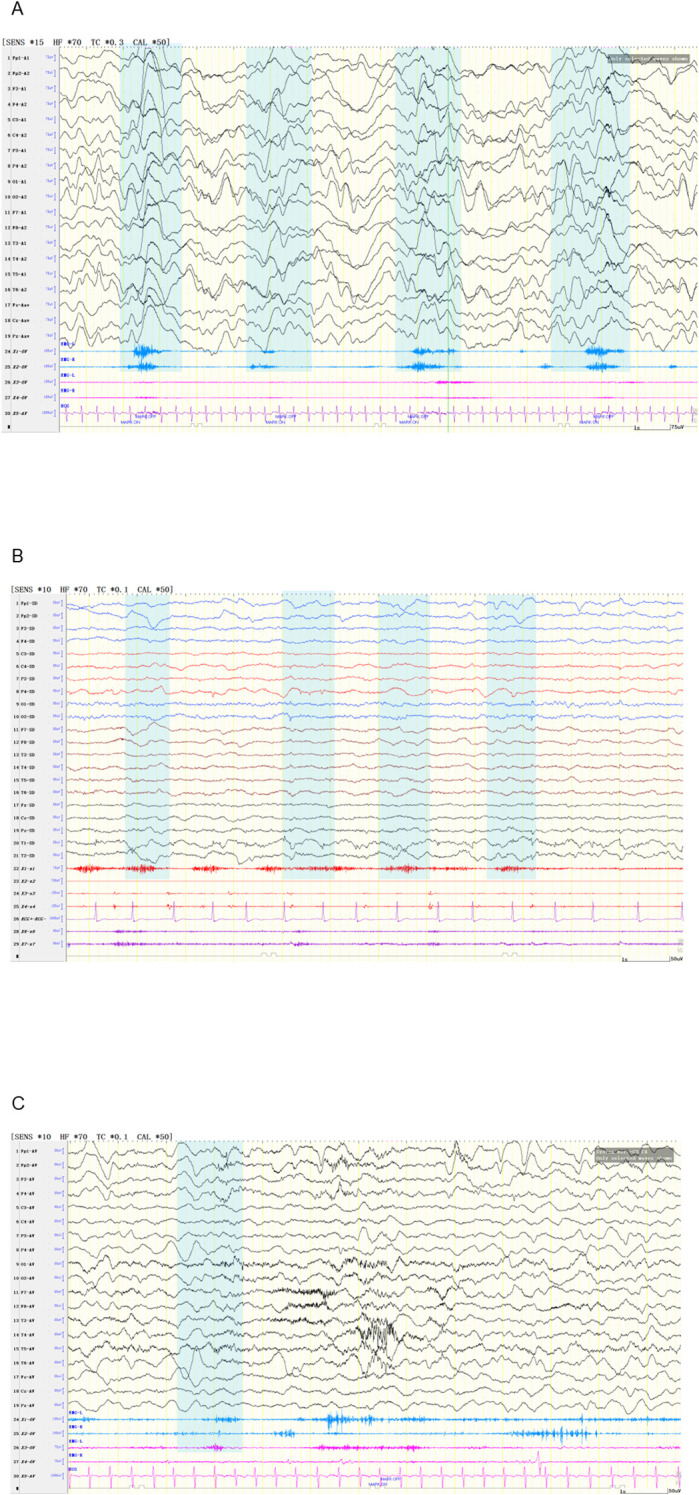
The electroencephalogram (EEG) manifestations of epileptic spasms in the three patients. **(A)** EEG of Case 1 showed diffuse medium-to-high amplitude slow waves combined with low amplitude fast waves when epileptic spasms. **(B)** EEG of Case 2 showed intermittent slow waves with a frequency of 1.5-3Hz and shaped slow waves in the left frontal, central and parietal regions when epileptic spasms. **(C)** EEG of Case 3 showed diffuse low-amplitude fast waves when epileptic spasms.

A 9-month-old boy was admitted our hospital with fever and seizure for half hour. His temperature had peaked at 38.5°C. Upon presentation, he developed status epilepticus at a body temperature of 37.7 °C, characterized by reduced consciousness, stiff and shaking rhythmic movements of the right limb, and hypersalivation. Occasionally, he had a little irritable. Physical examination revealed slightly plump of the anterior fontanelle, less movement and lower muscle tone of the right limb, and positive both Babinski signs. After admission, a lumbar puncture was performed. Cerebrospinal fluid (CSF) analysis showed a cell count of 6 ×10^6^/L, with normal protein and glucose levels. Anti-NMDAR antibodies were negative in both serum and CSF as assessed by cell-based assay. High-throughput sequencing of CSF identified herpes simplex virus type 1 (HSV-1), confirming active central nervous system infection with this pathogen. Electroencephalography (EEG) demonstrated abnormal discharges over the left central and superior regions and monitored a focal motor seizure (**Figure 1A**). Brain magnetic resonance imaging (MRI) revealed abnormal signals in the bilateral frontal lobes and left parietal lobe, suggesting inflammatory changes. A preliminary diagnosis of viral encephalitis was made. The patient received antiviral therapy along with the antiseizure medication (ASM) of levetiracetam (LEV). After two weeks of treatment, he became seizure-free, but experienced reduced flexibility in his right upper limb.

**Figure 2:**
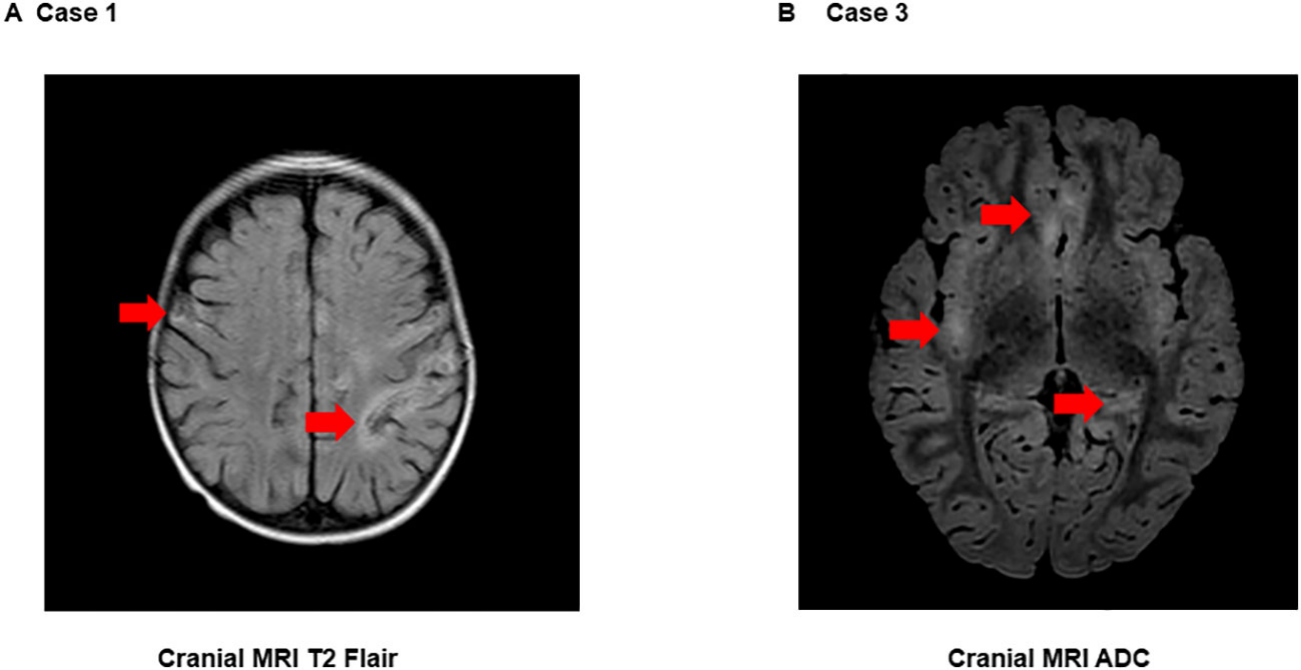
Brain magnetic resonance imaging (MRI) of Case 1 and Case 3. **(A)** Brain MRI of Case 1 showed multiple abnormal signals in the bilateral frontal lobes, left parietal lobe, and bilateral thalamus (as indicated by the red arrow). **(B)** Brain MRI of Case 3 showed multifocal abnormal signals involving the bilateral frontal lobes, insular cortices, and left temporal lobe (as indicated by the red arrow).

**Figure 3:**
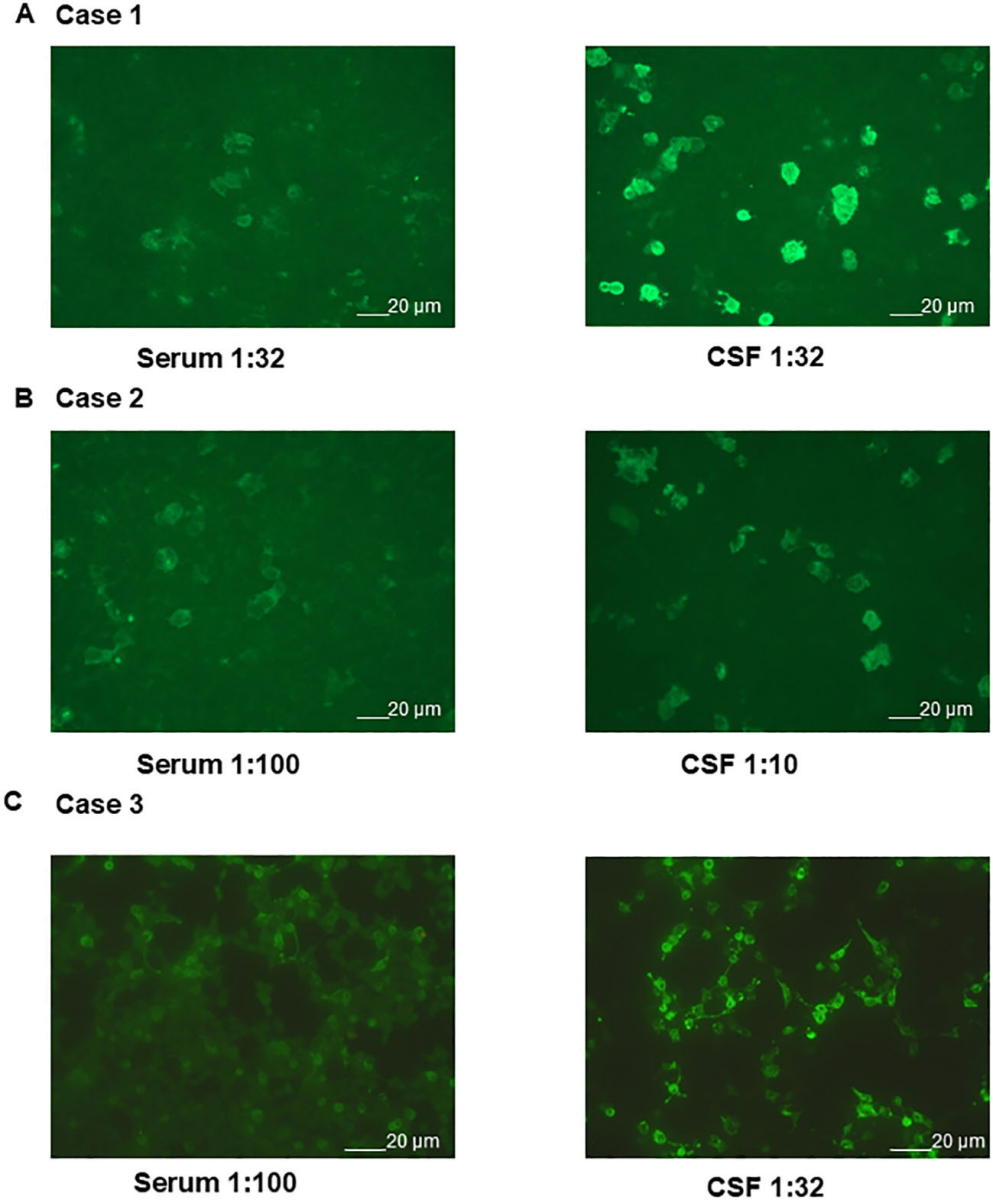
Anti-NMDAR antibodies titer of three patients. **(A)** NMDAR-IgG antibodies of Case 1 in Serum (1:32) and CSF (1:32). **(B)** NMDAR-IgG antibodies of Case 2 in Serum (1:100) and CSF (1:10). **(C)** NMDAR-IgG antibodies of Case 3 in Serum (1:100) and CSF (1:32).

However, a follow-up brain MRI revealed progression compared to before, showing multiple abnormal signals in the bilateral frontal lobes, left parietal lobe, and bilateral thalamus (**Figure 2A**), with areas of softening and cortical laminar necrosis. Meanwhile, the child experienced a recurrence of frequent seizures, followed by dysphagia, impaired consciousness, distortion of commissure, and recurrent fever. Due to the patient’s age being less than 1 year old, the mRS score is not applicable. A repeated LP revealed pleocytosis (24 ×10^6^/L) in the CSF, with normal protein and glucose level. Notably, NMDAR-IgG antibodies were detected by cell-based assay in both serum (1:32) and CSF (1:32) (**Figure 3A**). Pathogen examination of CSF returned negative results. EEG showed frequent epileptic spasms. Based on these findings, the diagnosis was anti-NMDAR encephalitis.

Despite receiving standard first-line immunotherapy of intravenous methylprednisolone (IVMP, 15mg/kg/day for 3 days, followed by a stepwise reduction, sequential oral prednisone administration) and intravenous immunoglobulin (IVIG, 0.5g/kg/day for 4 days), along with the addition of topiramate (TPM), the patient’s condition continued to deteriorate. He remained unconscious, with frequent seizures, occasional irritation, and recurrent fever. Owing to the aggressive course and communication with his parents, a second course of IVIG (0.5g/kg/day for 4 days) was administered; however, no clinical improvement was observed. Ultimately, his parents declined further intensive treatment and requested discharge due to financial difficulties and requested discharge. The child passed away during the 4−week post−discharge follow−up period.

### 2.2 Case 2

A 9 year-4-month-old girl was admitted our hospital with a one-week history of seizures and progressive aphasia. Her seizures manifested as status epilepticus in two distinct forms, one characterized by continuous twitching of the mouth with leftward deviation and shaking of the right upper limb; the other presenting as blank staring and unresponsiveness without limb movements, each lasting 5 to 20 minutes. Concurrently, she experienced gradually worsening aphasia. Initially, she presented reduced vocabulary, slowed speech initiation, and decreased speaking rate. Gradually, her comprehension became difficult, and by the time of admission she could only utter the word “Mom.” These episodes were often accompanied by involuntary movements of the right hand and significant irritability. The parents reported no recent history of infection and was in good health with normal development of child in the past. On admission, despite administering multiple conventional sedatives, her uncontrolled status epilepticus, progressive irritability, and conscious disturbances required intensive care unit (ICU) (mRS 5 score).

Physical examination showed bilaterally brisk patellar tendon reflexes, with no other neurological deficits. After admission, CSF analysis showed mild lymphocytic pleocytosis (15 × 10^6^/L) with normal protein and glucose levels. NMDAR-IgG antibodies were detected in serum (1:100) and CSF (1:10) by cell-based assay (**Figure 3B**). CSF pathogen examination was negative; however, serum testing was positive for HSV-1 IgE antibodies, rendering the pathogenic role of HSV-1 in this case uncertain. EEG monitored focal epileptic spasms arising from the left central and superior lobe, consistent with epilepsia partialis continua (EPC) (**Figure 1B**). Brain MRI showed unremarkable. Given the above evidences, a diagnosis of anti-NMDAR encephalitis was confirmed.

During the initial treatment in ICU, the patient received intensive immunotherapy including a 5- round course of therapeutic plasma exchange, IVMP (20 mg/kg/day for 3 days, followed by a stepwise tapering regimen), and continuous midazolam infusion for seizure control. Intravenous LEV was added for three days. Following this regimen, his consciousness gradually improved, responsiveness returned, and irritability lessened, although involuntary movements of the right hand persisted. After thorough discussion with the family, a course of IVIG (0.5 g/kg/day for 4 days) was administered, and perampanel (PER) was introduced. At the three- month follow- up, she had recovered significantly, with normal communication ability, no seizures, and resolution of involuntary movements (mRS 0 score).

However, she experienced a clinical recurrence of NMDARE at the 8−month follow−up. The manifestation of recurrence included recurrent aphasis, irritability, memory deterioration (mRS 3 score), and focal clonic seizures monitored by EEG. NMDAR-IgG antibodies were detected in serum (1:320) and CSF (1:32) by cell-based assay. After the combined treatments of IVIG (0.5g/kg/day for 4 days) for two rounds, IVMP (20mg/kg/day for 3 days, halved stepwise reduction), and rituximab, she gradually recovered well again. At the last follow-up of five months, she has basically returned to normal (mRS 0 score).

### 2.3 Case 3

A 5-year-2-month-old girl was admitted to our hospital with an 11-day history of sleep disturbance and lethargy, followed by one day of seizures. Her sleep disturbance was characterized by poor sleep quality, difficulty to fall sleep, somniloquy (sleep-talking), and irritability. The seizures manifested in two forms, one involved shaking of the shoulders and upper trunk accompanied by unresponsiveness for approximately 30 minutes; the other presented with eye deviation, cyanotic lips, generalized rigidity, and loss of consciousness, lasting 5–6 minutes (mRS 3 score). She had been previously healthy, and was the only child of non−consanguineous parents, both of whom were healthy. Her mother had an uncomplicated natural conception, pregnancy, and delivery. There was no history of perinatal asphyxia or other neonatal complications.

Physical examination revealed a positive right Babinski sign, with no other focal neurological deficits identified. After admission, CSF analysis demonstrated pleocytosis (73 × 10^6^/L) with normal protein and glucose levels. Anti-NMDAR antibodies were detected by cell−based assay in both serum (titer 1:100) and CSF (titer 1:32) (**Figure 3C**). CSF pathogen screening was negative; however, serum testing showed elevated HSV−1 IgE antibodies (index 1.71), rendering the pathogenic role of HSV-1 in this case uncertain. EEG showed frequent interictal epileptiform discharges and slow−wave activity over bilateral posterior regions. Brain MRI revealed multifocal abnormal signals involving the bilateral frontal lobes, insular cortices, and left temporal lobe (**Figure 2B**). Based on these findings, a definitive diagnosis of anti−NMDAR encephalitis was established.

Following the diagnosis, first−line immunotherapy was initiated with IVMP (20 mg/kg/day for 3 days, followed by stepwise tapering) and IVIG (0.4 g/kg/day for 5 days), along with a subsequent oral prednisone taper. PER was also administered for seizure control. Despite this regimen, the patient’s condition deteriorated rapidly. She exhibited worsening speech impairment and cognitive decline, accompanied by new−onset epileptic spasms and involuntary movements of the left upper limb (mRS 5 score). A repeat EEG revealed diffuse background slowing with abundant interictal epileptiform discharges over the bilateral posterior and centroparietal regions, and monitored clusters of epileptic spasms (**Figure 1C**).

Given the lack of improvement with first−line immunotherapy, second−line treatment was initiated, consisting of rituximab (375 mg/m² per dose for 4 doses) and tocilizumab (150 mg per dose for 1 dose). Meanwhile, administer clobazam (CLB) and ketogenic diet (KD) to combined antiseizure treatment. Upon the above combined treatment, she responded promptly with a complete resolution of seizure, especially in cognitive functions. Functionally, the patient regained swallowing ability and walk ability with support, although her speech rate remained slightly slow. Follow−up brain MRI showed abnormal signals in the brain have largely resolved compared to before. Thereafter, she received monthly IVIG (1–2 g/kg) for immune maintenance over six months. During this period, her recovery showed a noticeable improvement, with ability to walk without support and language ability as before (mRS 2 score).

## 3 Discussion

We reported three pediatric cases of NMDARE in which frequent epileptic spasms emerged during the acute phase, representing a distinct seizure type within the spectrum of NMDARE-related epilepsy. Although epileptic spasms are a well−recognized and often refractory seizure phenotype in infants and young children, their occurrence in the acute stage of pediatric NMDARE remains scarcely reported. This case series expanded the known phenotypic spectrum of the disease and offers clinically relevant insights to early recognition and management.

### 3.1 Possible mechanisms of epileptic spasms in NMDARE

In patients who develop epileptic spasms, neuronal hyperexcitability extends broadly, involving not only the cerebral cortices but also deep brain structures such as the cerebellum, brainstem, and deep gray matter (14). In Case 1, the 9−month−old infant had immature cerebral white matter and a prior episode of HSV−1 encephalitis, which resulted in multifocal lesions in the bilateral frontal lobes and left parietal lobe. The subsequent epileptic spasms in the setting of anti−NMDAR encephalitis may therefore be linked to these pre−existing structural injuries caused by viral infection. Similarly, in Case 3, structural etiology should be also considered. Her epileptic spasms emerged approximately 16 days after disease onset and were likely related to diffuse inflammatory brain injury incurred during the acute encephalitic phase. Epileptic spasms are known to occur in the context of post−encephalitic epilepsy, especially in patients with widespread cerebral damage and significant cognitive impairment (14, 15). The pathogenic role of HSV- 1 in Cases 2 and 3 remains uncertain and may represent an incidental finding or a trigger for autoimmunity, rather than active encephalitis. In contrast, Case 2 developed epileptic spasms 21 days into the illness without evidence of structural lesions on MRI. This finding strongly suggests that, in a subset of patients, epileptic spasms may arise from widespread functional network disruption during the acute phase of NMDARE, even in the complete absence of detectable structural lesions. This observation aligns with the well- recognized phenomenon that MRI is frequently normal in anti- NMDAR encephalitis, and further supports the notion that antibody- mediated synaptic dysfunction alone can generate severe epileptiform activity (14).

Indeed, prior study has indicated that the presence of structural lesions on MRI during the acute phase of NMDARE may correlate with better seizure control (16). Nevertheless, the relationship between MRI findings and the overall prognosis of patients with NMDARE and seizures has been controversial. While some investigators have proposed that abnormal MRI does not significantly alter the generally favorable long-term outcome of these patients (16, 17), more recent studies have suggested that brain MRI abnormalities in NMDARE may be associated with poorer functional recovery and a higher risk of disease recurrence (18, 19).

Mechanistically, anti-NMDAR antibodies target an epitope on the GluN1 subunit, leading to receptor internalization and a consequent decrease in synaptic NMDAR density, which underlies the reversible synaptic dysfunction characteristic of the disease (20). Immunotherapy reduces antibody titers, allowing receptor re−expression and functional recovery. However, distinct from this reversible internalization, persistent structural damage can occur through additional pathways, such as T−cell−mediated cytotoxicity, which is increasingly recognized in antibody−associated encephalitis (21). The neurophysiological basis of epileptic spasms in NMDARE remains unclear. Although not directly examined in our cases, emerging evidence suggests that they may arise from a convergence of mechanisms, including the acute, antibody- mediated synaptic dysfunction described above, combined with structural injury and epileptogenic network disruption that likely involves T- cell- driven cytotoxicity and diffuse cerebral irritation (20). We propose, as a hypothesis- generating framework, that this combined insult could lower the seizure threshold and facilitate the expression of spasms in susceptible developing circuits. However, we acknowledge that these mechanistic interpretations will require confirmation in future experimental or pathological studies.

### 3.2 Management of epileptic spasms in acute-phase NMDARE

During the acute phase, individualized treatment is necessary for the seizure outcomes. Some patients may achieve seizure- free status with or without ASMs, whereas others may progress to refractory epilepsy despite long- term ASMs (7). Currently, the treatment duration and selection of ASMs in the acute phase of NMDARE have been controversial, especially in the absence of clear guidelines. Some studies suggested that most patients with NMDARE do not require the use of ASMs, particularly for patients who have achieved seizure- free status after first-line treatments (7, 10). However, as a relatively refractory seizure type among the epilepsy group, epileptic spasms in pediatric NMDARE should be early treated by ASMs. And in clinical practice, we often prioritize AEDs with a relatively fast drug titration to control the seizures as soon as possible, especially for those with status epilepticus. In our cases, LEV (administered intravenous infusion for three days, then proceed with oral administration in sequence), PER, and KD (starting from dietary ratio 4:1) were the most common treatments of NMDARE. Specifically, we note that although the outcomes in our three cases were heterogeneous, the occurrence of epileptic spasms in the acute phase may suggest a tendency toward a more severe disease course. The favorable outcomes are still achievable with timely and intensive immunomodulation, as demonstrated by Case 3. Regarding the cessation of spasms, we observed that seizure control was closely associated with the response to immunotherapy: in Cases 2 and 3, epileptic spasms resolved following combined first- and second- line immunotherapy, whereas in Case 1, lack of response to treatment and subsequent withdrawal of care precluded meaningful assessment.

Given the mechanism of NMDARE and the importance of human gamma globulin treatment, ASMs alone cannot contribute to a good seizure outcome in the acute phase. Previous literatures recommended the combination of ASMs with immunotherapy (22). Therefore, an early combination of immunotherapy as the basic treatment and ASMs as an add-on treatment is optimal for pediatric NMDARE with epileptic spasms. From long-term perspective, previous study has shown that seizure freedom was achieved within two years in all patients with NMDARE, and more than 80% with acute seizures had their last seizure within six months of disease onset (10). Therefore, it is generally believed that long-term ASMs treatment for seizures presented in the acute phase of NMDARE may not be necessary (11). However, the treatment duration of ASMs for epileptic spasms still requires further research to be proven.

## 4 Conclusion

This case series identifies epileptic spasms as an acute-phase seizure phenotype in pediatric anti-NMDAR encephalitis. This presentation expands the known clinical spectrum of the disease and should alert clinicians to consider AE in children with acute encephalopathy and spasms. Timely recognition can prompt appropriate diagnostic workup and early immunotherapy, which is crucial for improving outcomes. Further studies are needed to define the prevalence and prognostic significance of this presentation.

## Data Availability

The original contributions presented in the study are included in the article/supplementary material. Further inquiries can be directed to the corresponding authors.
